# Coronavirus Disease 2019 Pandemic – Public Health Preparedness in Nepal and One Health Approach

**DOI:** 10.1017/dmp.2020.172

**Published:** 2020-05-29

**Authors:** Sunil Pokharel, Shristi Raut, Komal Raj Rijal, Bipin Adhikari

**Affiliations:** Centre for Tropical Medicine and Global Health, Nuffield Department of Medicine, University of Oxford, Oxford, United Kingdom; Department of Microbiology, Universal College of Medical Sciences, Bhairahawa, Nepal; Central Department of Microbiology Tribhuvan University, Kirtipur, Kathmandu, Nepal; Centre for Tropical Medicine and Global Health, Nuffield Department of Medicine, University of Oxford, Oxford, OX1 3SY, United Kingdom

**Keywords:** COVID-19, epidemics, low and middle income countries (LMICs), public health preparedness, one health

The coronavirus disease 2019 (COVID-19) pandemic that originated in Wuhan, China, in December 2019 has spread to 210 countries, infected more than 4 million people, and claimed 313 220 lives as of May 17, 2020.^1,2^ While high-income countries are struggling to control the disease, of the bigger concern is whether low- and middle-income countries (LMICs) with weak health systems have adequate capabilities to tackle the infection. Drawing on our perspective from Nepal, we outline the challenges and recommendations relevant for LMICs.

LMICs, like Nepal, have a high burden of poverty, diseases, and a poor health system infrastructure.^3^ Intervention measures, such as a complete lockdown, have apparently successfully delayed the spread of COVID-19, but they bear catastrophic socioeconomic consequences and are alone unsustainable.^4^ While an effective vaccine to generate sufficient herd immunity is not a solution for the foreseeable future, alternative strategies to contain the virus, *severe acute respiratory syndrome coronavirus 2 (SARS-CoV-2)*, based on robust surveillance data, are urgently required. Strengthening health system capacity focused on sustainable COVID-19 preparedness and targeted interventions with extended diagnostic testing, contact tracing, and enhanced capacity for early isolation and treatment of cases are imperative for future containment efforts.

LMICs lack adequate diagnostic infrastructure. A central lab in Kathmandu and few provincial labs in Nepal with limited testing capacity for reverse transcriptase polymerase chain reaction (RT PCR) and poor capacity for sample chain logistics for testing from the peripheral health facilities, impeded by poor network and transportation infrastructure, are unlikely to manage the timely diagnosis and response.^4^ The available rapid test for disease screening has low specificity for active infection and offers little help for diagnosis and clinical monitoring.

The current hospital preparedness of Nepal is constrained by inadequate isolation rooms, intensive care unit facilities, and ventilators limited to urban settings.^5^ In addition, Nepal, like other LMICs, suffer from high syndemic burden of infectious diseases, which can compound the diagnosis and strain the existing scarce health care resources. In addition, high comorbidities may simply offer a sense of complacency for the justification of high mortalities. Nepal has been struggling with inadequate personal protective equipment for the frontline health care workers, which has increased their vulnerability to infection.^6^

Measures for quarantine and isolation have prompted fear, discrimination, and rejection among the population in Nepal.^7^ Fear and stigmatization are recognized barriers to early health seeking and hinder early case identification – perpetuating community transmission.^8^ Linked to fear and stigmatization, hyped up, uncensored, and unverified news in both formal and informal media can have an adverse impact on social harmony.^9^ Urgent actions toward censoring the fake news, rumors, and unproven messages in both formal and informal media require strict scrutiny and regulation.

Although the transmission of SARS-CoV-2 from animals to human has not yet been established, the identification of this virus from both domestic and wild animals demands further precautionary endeavors.^10^ In addition, reports of the dissemination of the virus in human excreta^11^ and the long survival of the virus in the environment institute it as a strong reservoir for the circulation of the virus.^12^ The control efforts should encompass protection of domestic animals and environmental hygiene. One health surveillance of SARS-CoV-2 is needed for early detection of this virus in animal and the environment to prevent transmission in communities.

Human species are non-immune to the huge reservoir of circulating pathogens in wildlife, and the consequent (re-) emerging zoonotic infections pose the highest pandemic potential.^13^ Outbreaks of coronavirus infections, including the SARS outbreak in 2003 and Middle East respiratory syndrome (MERS) outbreaks in 2012, 2015, and 2018, and the recent COVID-19 outbreak, have been associated with the zoonotic reservoirs.^14,15^ Hunting, trade, and consumption of wild animals such as monkeys, rats, bats and snakes in many LMICs, including Nepal, pose a significant threat of introducing zoonotic diseases to the human ecosystem. Measures toward adopting “one health approach” and change in evolving food behavior directed toward exotic and wild animal foods are essential in mitigating the risk of future epidemics.^13^
